# Obstetric Outcomes by Hospital Volume of Operative Vaginal Delivery

**DOI:** 10.1001/jamanetworkopen.2024.53292

**Published:** 2025-01-06

**Authors:** Annika S. Willy, Alyssa R. Hersh, Bharti Garg, Aaron B. Caughey

**Affiliations:** 1 Department of Obstetrics & Gynecology, Oregon Health & Science University, Portland

## Abstract

**Importance:**

Characterizing hospital-level factors associated with adverse outcomes following operative vaginal delivery (OVD) is crucial for optimizing obstetric care.

**Objective:**

To assess the association between hospital OVD volume and adverse outcomes.

**Design, Setting, and Participants:**

This was a retrospective cohort study of OVDs in California between 2008 and 2020. OVD was determined using birth certificate and *International Classification of Diseases, Ninth Revision, Clinical Modification* or *International Statistical Classification of Diseases, Tenth Revision, Clinical Modification* codes. This study used linked vital statistics and hospital discharge data from California. The study included singleton, nonanomalous, full-term deliveries with vertex presentation. Data analysis was performed between June 10 and October 23, 2024.

**Exposure:**

Hospital OVD volume was categorized by the proportion of OVDs performed among all deliveries, grouped into low (<5.2%), medium (5.2%-7.4%), and high (≥7.4%) volume.

**Main Outcomes and Measures:**

Adverse outcomes for birthing individuals included obstetric anal sphincter injuries, cervical lacerations, and postpartum hemorrhage. Neonatal outcomes included shoulder dystocia, subgaleal hemorrhage, intracranial hemorrhage, facial nerve injury, and brachial plexus injury (BPI). χ^2^ and multivariable Poisson regression analyses were used to assess the association between hospital OVD volume and outcomes.

**Results:**

Among 306 818 OVDs (mean [SD] birthing parent’s age, 28.5 [6.2] years; 155 157 patients with public insurance [50.6%]), hospitals with low OVD volume had an increased proportion of obstetric anal sphincter injury compared with hospitals with medium and high volumes (12.16% [7444 patients] vs 11.07% [10 709 patients] vs 9.45% [14 064 patients]). Hospitals with low volume also had a higher proportion of adverse neonatal outcomes, including shoulder dystocia (3.84% [2351 patients] vs 3.50% [3386 patients] vs 2.80% [4160 patients]), subgaleal hemorrhage (0.27% [165 patients] vs 0.18% [172 patients] vs 0.10% [144 patients]), and BPI (0.41% [251 patients] vs 0.30% [291 patients] vs 0.20% [301 patients]) compared with hospitals with medium and high volume. In multivariable analyses, low OVD volume remained associated with increased risk of obstetric anal sphincter injury (adjusted risk ratio [aRR], 1.36; 95% CI, 1.14-1.62), shoulder dystocia (aRR, 1.30; 95% CI, 1.10-1.52), subgaleal hemorrhage (aRR, 2.57; 95% CI, 1.55-4.24), and BPI (aRR, 1.73; 95% CI, 1.30-2.2.29) compared with hospitals with high OVD volume. After multivariable analysis, medium OVD volume remained associated with increased risk of subgaleal hemorrhage (aRR, 1.72; 95% CI, 1.04-2.86) and BPI (aRR, 1.35; 95% CI, 1.02-1.79) compared with high OVD volume.

**Conclusions and Relevance:**

This study found that undergoing OVD at hospitals with low OVD volume was associated with adverse perinatal outcomes compared with hospitals with medium and high OVD volumes. Further exploration of the reasons for these differences and prevention of these differences is needed to improve obstetric outcomes.

## Key Points

**Question:**

Is hospital volume of operative vaginal deliveries (OVDs) associated with perinatal outcomes of OVD?

**Findings:**

This cohort study included 306 818 newborns delivered via OVD. Hospitals with low OVD volume had significantly higher proportions of newborns with shoulder dystocia, neonatal intensive care unit admissions, subgaleal hemorrhage, and brachial plexus injury compared with hospitals with medium and high OVD volumes.

**Meaning:**

These findings suggest that hospitals with low OVD volume had higher rates of adverse perinatal outcomes than those with medium and high volumes, and additional research into the reasons for these differences is needed to improve obstetric outcomes.

## Teaser

This cohort study examines the association between hospital operative vaginal delivery volume and adverse outcomes among newborns and their birthing parents.

## Introduction

In modern obstetric practice, the mode of delivery plays a pivotal role in outcomes among birthing parents and neonates. Operative vaginal delivery (OVD) refers to vaginal deliveries in which forceps or vacuum devices are used to facilitate delivery when clinically indicated. Common indications for OVD include protracted second stage of labor, fetal compromise, and the birthing parent’s medical conditions.^1,2^ OVD may provide a safe alternative to cesarean delivery in cases where clinician intervention is required.^3^ Given that risks from cesarean delivery include longer hospital stay, more difficult recovery, and greater potential for complications such as infection, as well as increasing risks to future pregnancies, including the risks of additional cesarean deliveries, placenta accreta, stillbirth, and uterine rupture, avoiding a cesarean delivery in a first pregnancy is preferred.^4,5,6,7^

The rate of OVD varies across the globe and over time.^8,9^ In the US, the rate declined from 9.4% in 1994 to 3% in 2014.^8,10^ In contrast, the rate of OVD among European countries over a similar 20-year period increased from 14% in 1991 to 23% in 2010.^11,12^ Since its decline to 3%, the OVD rate in the US has remained stable, with an OVD rate of 3.04% in 2020.^13^ As of 2021, rates from Europe showed variation across countries, from 13% in England, to 12.4% in France, and 13.5% in Ireland.^14,15,16^ In the US, this decrease in OVD use has coincided with increasing rates of cesarean deliveries, from 22% in 1994 to 31.8% in 2020,^13,17^ although the increase in cesarean deliveries cannot be attributed solely to the decrease in OVD rates, because other factors such as primary cesarean delivery elected by the birthing individual, the birthing individual’s comorbidities, and multiple gestation have contributed to the increase in cesarean deliveries.^7,18^ In addition, the first cesarean delivery increasing the likelihood of cesarean delivery in future pregnancies has a compounding effect on the cesarean delivery rate.^18^ Prior studies^19^ have found that hospitals with high rates of OVD have low rates of cesarean delivery compared with hospitals with low OVD rates. Given that OVD is a safe alternative to cesarean delivery for the aforementioned indications, it is unclear what is leading to the decrease in OVD.^3^ Hypothesized reasons for decreasing rates of OVD include lack of clinician comfort and experience, patient preference, and concern for litigation.^8,9,20^ Fear of litigation and adverse outcomes from difficult vaginal deliveries are central factors associated with clinicians’ choosing cesarean deliveries.^21^ In addition, it is hypothesized that perceived worse outcomes in OVDs contribute to this hesitation; however, some studies^22,23,24^ suggest that the indication for OVD, rather than the procedure itself, plays a larger role in determining morbidity.

The association between hospital volume and patient outcomes has been well explored in prior literature. Higher rates of adverse outcomes in low-volume settings are well documented in both the surgical and medical literature.^25,26,27^ In obstetrics-specific research, hospitals with high volume of deliveries were found to have fewer adverse outcomes among birthing individuals compared with their low-volume and medium-volume counterparts.^28,29,30,31^ When investigating asphyxia and neonatal death by hospital volume in a low-risk population, neonatal death and asphyxia decreased from 18 cases per 10 000 live births at low-volume hospitals to 9 cases per 10 000 live births in high-volume hospitals.^31^ There is less current literature on the association between individual physician volume and patient outcomes, although studies^32,33^ have shown that surgeons with increased annual volume have decreased short-term adverse outcomes among birthing individuals following cesarean delivery.

Across the US among different hospitals, there are substantial differences in the rate of OVD.^34^ Understanding this variation in OVD outcomes across hospitals with different OVD rates is essential for evaluating the quality of obstetric care and identifying factors associated with delivery outcomes. As the rates of OVD decline in the US and considering the importance of maintaining proficiency in medical procedures through regular practice, it becomes imperative to investigate how variation in OVD rates is associated with overall obstetric outcomes. Therefore, this study aims to explore the association between hospital-specific OVD volume and the resultant perinatal outcomes, to help inform evidence-based practices in obstetric care.

## Methods

This was a retrospective, population-based, cohort study using linked vital statistics and hospital discharge data in California (2008-2020). Initial data analysis was completed on June 10, 2024, and was updated on October 23, 2024. We linked vital statistics data (birth and fetal death certificates) maintained by the California Department of Public Health to patient discharge data maintained by the Department of Health Care Access and Information; linkage was done at Oregon Health & Science University. Probabilistic linkage was used to link the 2 datasets; it used a combination of available common data elements and variables that were present in both datasets, such as neonatal birth date, neonatal sex, birth hospital, patients’ residential zip code, and hospital county. Of the 6 203 800 birth certificate records, 94.2% of the vital statistics data were linked to both parental and neonatal hospital discharge records. A comparison of the patients in the analytic sample and those who were excluded because of nonlinkage is provided in eTable 1 in Supplement 1. The final dataset included all birthing individuals’ and neonatal characteristics derived from birth and death certificates, as well as diagnosis and procedure codes recorded using *International Classification of Diseases, Ninth Revision, Clinical Modification (ICD-9-CM)* (January 2008 to October 2015) and *International Statistical Classification of Diseases, Tenth Revision, Clinical Modification (ICD-10-CM)* (October 2015 to December 2020) from hospital discharge records. This study was approved by the institutional review board at Oregon Health & Science University and Committee for the Protection of Human Subjects of the Health and Human Services Agency in the State of California. Informed consent was not required because the data were deidentified and publicly available, in accordance with 45 CFR §46. This study followed the reporting requirements of the Strengthening the Reporting of Observational Studies in Epidemiology (STROBE) reporting guidelines.^35^

We included pregnant individuals with singleton, nonanomalous, full-term (37-42 weeks) OVDs. Exclusion criteria were multifetal gestation, anomalous births, presentation other than vertex, and gestational ages less than 37 weeks and more than 42 weeks. We further excluded patients with spontaneous vaginal deliveries, cesarean deliveries, or unknown mode of delivery. Hospital OVD volume was estimated as the proportion of OVDs among all deliveries in each hospital during the study period. Hospitals were then divided into tertiles on the basis of the OVD rate at each hospital. We believe these rates are closely associated with physician volumes. Tertiles included low OVD volume (<5.2%), medium OVD volume (5.2%-7.4%) and high OVD volume (≥7.4%); these rates were the demarcations that divided the hospitals into 3 tertiles, with each tertile having a similar total number of deliveries. These rates represent the percentage of all deliveries that were OVD at each hospital. Of 274 hospitals in California, 96 had low OVD volume, 90 had medium volume, and 88 had high volume. We included all OVDs to examine the association of hospital’s OVD volume with adverse parental and neonatal outcomes among patients with OVD. OVD as mode of delivery was determined using birth certificate, *ICD-9-CM* codes and *ICD-10-CM* codes (eTable 2 in Supplement 1).

Demographics of birthing individuals were retrieved from birth certificate or hospital discharge data, and included race and ethnicity, age, parity, education, insurance, prepregnancy body mass index (BMI; calculated as weight in kilograms divided by height in meters squared), gestational age, and birth weight. Birthing parents’ age was grouped into 3 categories (<20, 20-34, or ≥35 years). Parity was defined as nulliparous or multiparous. Self-reported race and ethnicity were classified into 1 variable as American Indian or Alaska Native, Asian, Hispanic, non-Hispanic Black, non-Hispanic White, and other (ie, participants selected “other”) or multiracial (ie, patients reported >1 race). Data on race and ethnicity are included in this study to help address the disparities in health care access and outcomes experienced by different racial and ethnic groups, recognizing that these differences are shaped by systemic and social factors rather than inherent biological differences. The birthing parent’s educational attainment was stratified as attendance through high school or less compared with having attended some college. Insurance status was grouped as private vs public, self-pay, and uninsured. Prepregnancy BMI was tabulated as underweight (<18.5), normal weight (18.5-24.9), overweight (25.0-29.9), or obese (≥30.0).

Adverse outcomes were defined using *ICD-9-CM* codes, *ICD-10-CM* codes, or birth certificate data. The birthing parent’s outcomes of interest included obstetric anal sphincter injuries, cervical lacerations, and postpartum hemorrhage. Neonatal outcomes of interest included shoulder dystocia, neonatal intensive care unit (NICU) admission, subgaleal hemorrhage, intracranial hemorrhage, facial nerve injury, fractures (skull, humerus, or clavicular fractures), and brachial plexus injury. The source of these outcomes is described in eTable 2 in Supplement 1.

We had a small amount of missing data on covariates, including race and ethnicity (0.04%), prepregnancy BMI (3.87%), insurance status (0.002%), and parity (0.03%). We addressed these missing data by performing multiple imputation using chained equations to generate 20 imputed datasets prior to conducting regression analyses. Pooled estimates were performed according to the Rubin rule.^36^

### Statistical Analysis

Comparisons of demographics and perinatal outcomes among low-volume, medium-volume, and high-volume OVD hospitals were performed using χ^2^ tests (categorical variables) and independent 2-sample *t* tests (continuous variables). Multivariable Poisson regression models were used to assess the association between hospital OVD volume and adverse outcomes, and adjusted risk ratios (aRRs) were estimated. The regression models evaluated the risk of perinatal outcomes among hospitals with low and medium OVD volume with reference to high OVD volume. Potential confounders included birthing parent’s age, race and ethnicity, education, prepregnancy BMI, parity, health insurance, birth weight, gestational age, year of delivery, total delivery volume, rural vs urban hospital, and delivery in academic hospital vs community hospital. All models were clustered on hospitals to reflect our analysis on hospital level. To check the robustness of our results, we performed sensitivity analysis using only birth certificate and then only *ICD-9-CM* or *ICD-10-CM* codes (eTable 3 in Supplement 1). We also examined the association of hospital volume with perinatal outcomes among patients with forceps and vacuum deliveries (eTable 4 in Supplement 1). An additional stratified analysis was done among nulliparous and multiparous patients (eTable 5 in Supplement 1). Statistical significance was set at 2-sided *P* < .05, and statistical analyses were performed using Stata statistical software version 18 (StataCorp).

## Results

**Figure 1.**
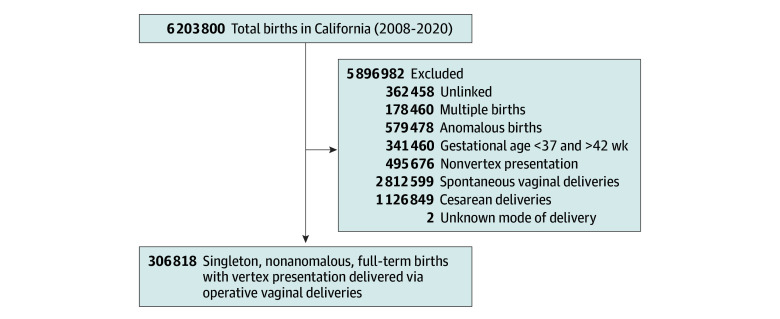
Participant Enrollment Flowchart

There were 306 818 patients (mean [SD] birthing parent’s age, 28.5 [6.2] years; 155 157 patients with public insurance [50.6%]) with OVD who met the inclusion criteria; 61 229 OVDs (19.9%) occurred in hospitals with low OVD volume, 96 771 (31.5%) occurred in hospitals with medium OVD volume, and 148 818 (48.5%) occurred in hospitals with high OVD volume (Figure 1). A higher proportion of the pregnant individuals with OVD in hospitals with low OVD volume were Hispanic (50.6% [30 973 patients] vs 37.9% [36 663 patients] vs 40.8% [60 660 patients]), were younger than 20 years (10.7% [6560 patients] vs 8.0% [7786 patients] vs 7.5% [11 098 patients]), and had obesity (16.4% [9647 patients] vs 13.2% [12 351 patients] vs 12.2% [17 371 patients]) compared with individuals in hospitals with medium and high OVD volume (Table 1).

**Table 1.** Demographics of Participants With OVD in California (2008-2020) According to Hospital OVD Volume

Characteristic	Patients, No. (%)			*P* value^a^
	Low volume (n = 61 229)	Medium volume (n = 96 771)	High volume (n = 148 818)	
Race and ethnicity				
American Indian or Alaskan Native (non-Hispanic)	180 (0.3)	262 (0.3)	222 (0.1)	<.001
Asian or Pacific Islander (non-Hispanic)	7170 (11.7)	16 893 (17.5)	35 679 (24.0)	
Black (non-Hispanic)	3075 (5.0)	3561 (3.7)	4093 (2.8)	
Hispanic	30 973 (50.6)	36 663 (37.9)	60 660 (40.8)	
White (non-Hispanic)	15 630 (25.5)	31 748 (32.8)	37 238 (25.0)	
Other or multiracial^b^	4189 (6.8)	7598 (7.9)	10 860 (7.3)	
Birthing parent’s age, y				
<20	6560 (10.7)	7786 (8.0)	11 098 (7.5)	<.001
20-34	45 243 (73.9)	71 466 (73.9)	110 115 (74.0)	
≥35	9426 (15.4)	17 519 (18.1)	27 605 (18.5)	
Prepregnancy body mass index^c^				
Underweight (<18.5)	2836 (4.8)	5504 (5.9)	10 418 (7.3)	<.001
Normal weight (18.5-24.9)	31 000 (52.7)	54 211 (58.1)	84 291 (59.0)	
Overweight (25.0-29.9)	15 303 (26.0)	21 295 (22.8)	30 702 (21.5)	
Obesity (≥30.0)	9647 (16.4)	12 351 (13.2)	17 371 (12.2)	
Some college education	33 762 (55.1)	63 740 (65.9)	96 296 (64.7)	<.001
Public insurance	31 554 (51.5)	42 790 (44.2)	80 813 (54.3)	<.001
Nulliparous	40 356 (65.9)	64 921 (67.1)	97 648 (65.6)	<.001
Birth weight, g	3361.6 (434.3)	3372.5 (432.5)	3359.8 (419.6)	<.001
Gestational age, mean (SD), wk	39.3 (1.1)	39.3 (1.1)	39.2 (1.0)	<.001
Total delivery volume, mean (SD), No. of deliveries	36 405.4 (20 240.0)	35 359.7 (19 130.4)	36 648.4 (22 360.9)	<.001
Rural hospital	2164 (3.5)	4541 (4.7)	7273 (4.9)	<.001
Academic hospitals	22 061 (36.0)	32 865 (34.0)	32 695 (22.0)	<.001

Abbreviation: OVD, operative vaginal delivery.

^a^ Calculated with χ^2^ test or 2-sample *t* tests.

^b^ Other includes participants who selected other, and multiracial includes participants who selected multiple races and ethnicities.

^c^ Body mass index is calculated as weight in kilograms divided by height in meters squared.

### Birthing Individuals’ Outcomes

**Figure 2.**
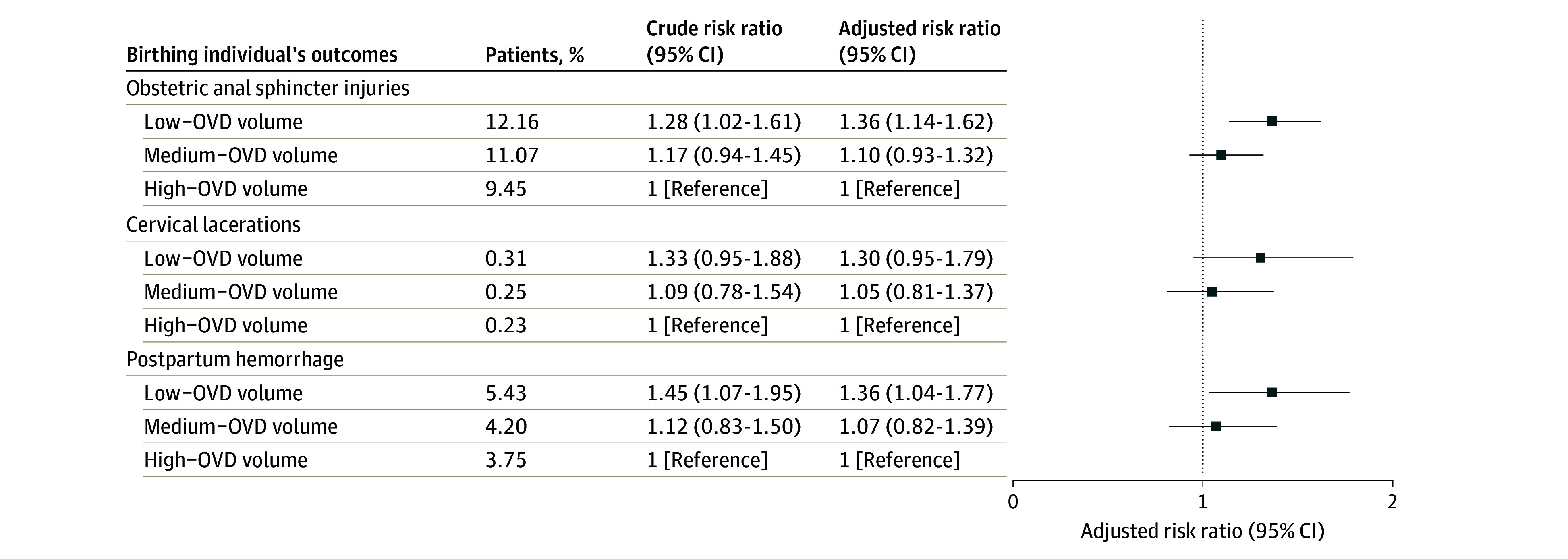
Association of Hospital Operative Vaginal Delivery (OVD) Volume With Adverse Outcomes Among Birthing Individuals Forest plot shows results of multivariable Poisson regression. Risk ratios were adjusted for birthing individual’s age, race and ethnicity, education, prepregnancy body mass index, parity, health insurance, birth weight, gestational age, year of delivery, total delivery volume, rural vs urban hospital, and academic vs community hospital.

Patients who delivered in hospitals with low OVD volume had a higher proportion of obstetric anal sphincter injuries (12.16% [7444 patients] vs 11.07% [10 709 patients] vs 9.45% [14 064 patients]), cervical lacerations (0.31% [189 patients] vs 0.25% [245 patients] vs 0.23% [343 patients]), and postpartum hemorrhage (5.43% [3325 patients] vs 4.20% [4069 patients] vs 3.75% [5586 patients]) compared with those who delivered at hospitals with medium and high OVD volume (Table 2). After adjusting for age, race and ethnicity, education, prepregnancy BMI, health insurance, parity, gestational age, neonatal birth weight, year of delivery, total delivery volume, rural hospitals, and academic hospitals, the risks of obstetric anal sphincter injuries (aRR, 1.36; 95% CI, 1.14-1.62) and postpartum hemorrhage (aRR, 1.36; 95% CI, 1.04-1.77) were significantly higher in hospitals with low OVD volume vs hospitals with high OVD volume (Figure 2). It is notable that although the adjusted risk of cervical lacerations (aRR 1.30; 95% CI 0.95-1.79) was not statistically significant, it was similar to the aRR of obstetric and sphincter injuries, supporting the validity of this finding.^37^

**Table 2.** Outcomes of Birthing Parents and Neonates by Hospital Operative Vaginal Delivery Volume in California (2008-2020)

Outcome	Patients, No. (%)			*P* value^a^
	Low volume (n = 61 229)	Medium volume (n = 96 771)	High volume (n = 148 818)	
Birthing parent’s outcomes				
Obstetric anal sphincter lacerations	7444 (12.16)	10 709 (11.07)	14 064 (9.45)	<.001
Cervical lacerations	189 (0.31)	245 (0.25)	343 (0.23)	.005
Postpartum hemorrhage	3325 (5.43)	4069 (4.20)	5586 (3.75)	<.001
Neonatal outcomes				
Shoulder dystocia	2351 (3.84)	3386 (3.50)	4160 (2.80)	<.001
Neonatal intensive care unit admission	6179 (10.09)	9030 (9.33)	12 985 (8.73)	<.001
Subgaleal hemorrhage	165 (0.27)	172 (0.18)	144 (0.10)	<.001
Intracranial hemorrhage	13 (0.02)	38 (0.04)	39 (0.03)	.08
Facial nerve palsy	30 (0.05)	28 (0.03)	39 (0.03)	.02
Fracture	326 (0.53)	474 (0.49)	555 (0.37)	<.001
Brachial plexus injury	251 (0.41)	291 (0.30)	301 (0.20)	<.001

^a^ Calculated with χ^2^ test.

### Neonatal Outcomes

**Figure 3.**
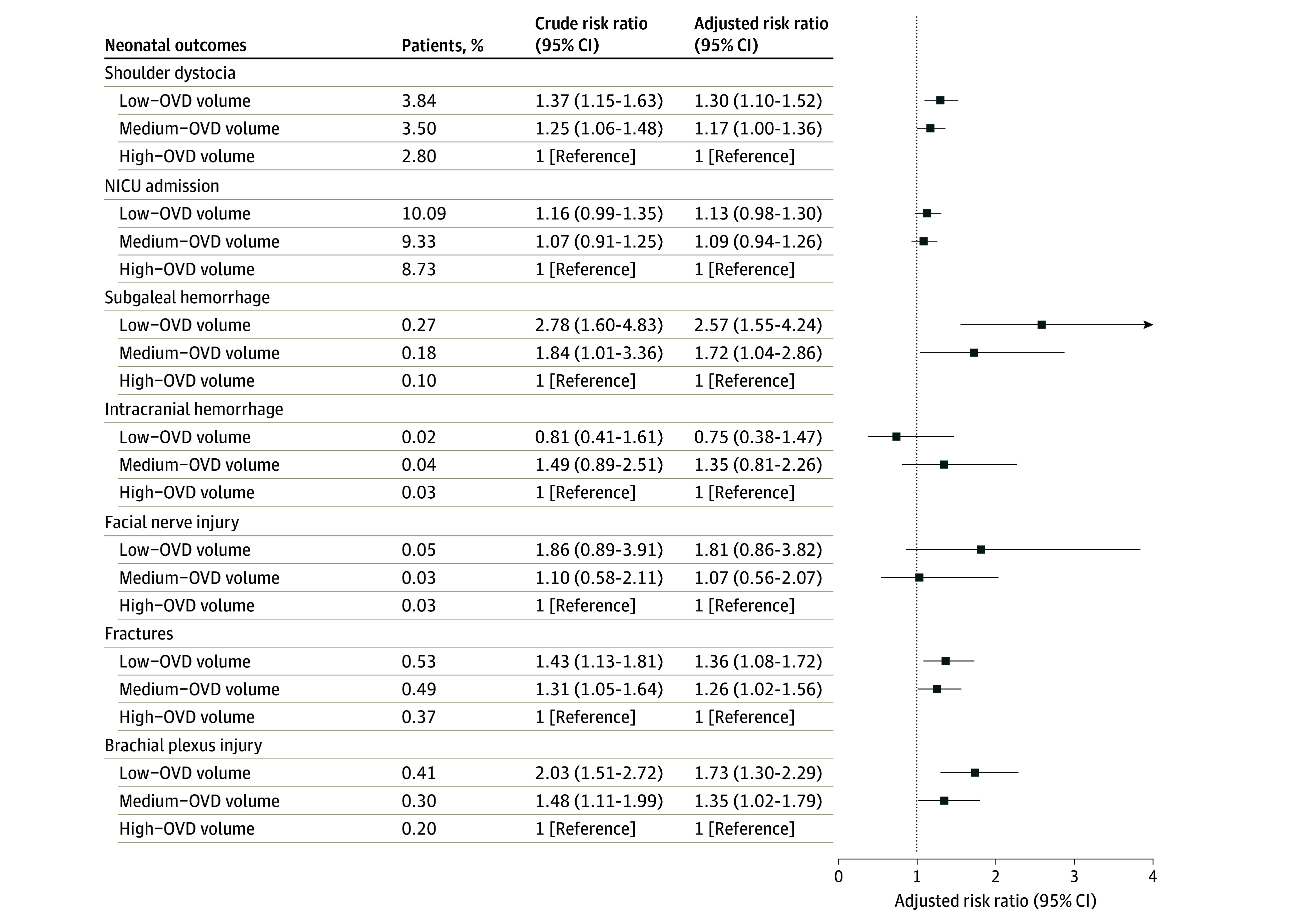
Association of Hospital Operative Vaginal Delivery (OVD) Volume With Adverse Neonatal Outcomes Forest plot shows results of multivariable Poisson regression. Risk ratios were adjusted for birthing parent’s age, race and ethnicity, education, prepregnancy body mass index, parity, health insurance, birth weight, gestational age, year of delivery, total delivery volume, rural vs urban hospital, and academic vs community hospital. NICU indicates neonatal intensive care unit.

Neonates delivered in hospitals with low OVD volume had higher proportions of shoulder dystocia (3.84% [2351 patients] vs 3.50% [3386 patients] vs 2.80% [4160 patients]), NICU admissions (10.09% [6179 patients] vs 9.33% [9030 patients] vs 8.73% [12 985 patients]), subgaleal hemorrhage (0.27% [165 patients] vs 0.18% [172 patients] vs 0.10% [144 patients]), facial nerve injury (0.05% [30 patients] vs 0.03% [28 patients] vs 0.03% [39 patients]), fractures (0.53% [326 patients] vs 0.49% [474 patients] vs 0.37% [555 patients]), and brachial plexus injury (0.41% [251 patients] vs 0.30% [291 patients] vs 0.20% [301 patients]) compared with those delivered at hospitals with medium and high OVD volume (Table 2). After adjusting for potential confounders, there were higher relative risks of shoulder dystocia (aRR, 1.30; 95% CI, 1.10-1.52), subgaleal hemorrhage (aRR, 2.57; 95% CI, 1.55-4.24), fractures (aRR, 1.36; 95% CI, 1.08-1.72), and brachial plexus injury (aRR, 1.73; 95% CI, 1.30-2.39) for hospitals with low OVD volume compared with high OVD volume. Hospitals with medium OVD volume had higher relative risks of fractures (aRR, 1.26; 95% CI, 1.02-1.56), subgaleal hemorrhage (aRR, 1.72; 95% CI, 1.04-2.86), and brachial plexus injury (aRR, 1.35; 95% CI, 1.02-1.79) compared with high OVD volume (Figure 3). In stratified analyses by birth certificate or *ICD-9-CM* or *ICD-10-CM* code (eTable 3 in Supplement 1), OVD type, forceps or vacuum (eTable 4 in Supplement 1) and by parity (eTable 5 in Supplement 1), results were generally in the same direction, but with loss of precision.

## Discussion

In this cohort study, we found significant disparities in perinatal outcomes among patients who delivered via OVD in hospitals of varying OVD volume categories. Delivering via OVD at a hospital with low OVD volume was associated with higher prevalence of adverse outcomes for birthing individuals, including obstetric anal sphincter injuries, cervical lacerations, and postpartum hemorrhage. Even after adjusting for various confounders, the risk of experiencing obstetric anal sphincter injuries and postpartum hemorrhage remained significantly higher in low OVD volume hospitals compared with high OVD volume hospitals. These findings underscore the importance of investigating the factors contributing to the differences in outcomes among OVDs and the need for potential quality improvement efforts to be undertaken to ensure optimal outcomes in the setting of OVDs in all settings.

In addition, our study highlights concerning trends in neonatal outcomes associated with OVD volume categories. Infants born in hospitals with low OVD volume had higher proportions of shoulder dystocia, NICU admissions, subgaleal hemorrhage, facial nerve injury, fractures, and brachial plexus injury compared with those born in hospitals with high OVD volume. After adjusting for confounders, being born in hospitals with low OVD volume was still associated with increased risk of shoulder dystocia, subgaleal hemorrhage, fractures, and brachial plexus injury. These findings underscore the importance of further research and interventions aimed at improving neonatal outcomes, particularly in low-volume settings.

Further research should analyze individual physician volume of OVD and obstetric outcomes. Although prior studies have attempted to analyze physician volume, many were limited to tertiary care centers and, ultimately, were underpowered regarding neonatal outcomes.^38^ Better understanding of how a physician’s annual or lifetime number of OVDs is associated with outcomes could be informative to understanding how to maintain skills and improve outcomes via quality improvement efforts. In addition to understanding the volume physicians need to maintain proficiency, additional investigation into the specific factors associated with outcome differences in hospitals with differing OVD volumes should be conducted.

### Limitations

Our study has inherent limitations that should be acknowledged. The utilization of administrative data, collected primarily for nonresearch purposes, introduces variability in reliability and accuracy. Nevertheless, in situations where randomization is impractical and extensive sample sizes are necessary, leveraging administrative data remains a pragmatic choice. Despite California’s substantial size and racial diversity, caution should be exercised in generalizing our findings to regions with distinct hospital policies, obstetric practices, and demographic compositions. In addition, the dataset did not include variables such as the hospital’s trauma level, NICU size and level, physician type and experience, and birthing parent and fetal medicine coverage, all of which are potential confounders. Furthermore, we used hospital-level proportions of OVD to stratify our sample into low-volume, medium-volume, and high-volume hospitals. Given that the proportion of OVDs at each hospital was determined as an average over the study period, it is possible that hospitals could have changed tertile over the course of the study period. However, such migration of hospital designation would have led to misclassification bias, which is toward the null, suggesting that our positive findings would persist. Also notable during the study period was the COVID-19 pandemic, which could have impacted these findings.

Furthermore, although our OVD proportion method may be reflective of individual physician volumes, it is not a replacement for having individual physician rates or individual physician total volumes. Without the individual physician information, it is not possible to determine the experience level of the practitioners at each facility. However, one would think that examining this issue at the hospital level would only bias findings toward the null and may also reflect the overall hospital environment in its receptivity and experience of OVDs.

## Conclusions

In this cohort study of California births, we found an association between hospital OVD volume and outcomes for birthing individuals and neonates. The risks of several perinatal outcomes were higher among OVDs performed at hospitals with low OVD volume. These findings suggest further investigation is needed to determine why hospitals with low OVD volume had higher risks of adverse outcomes, and what can be done to mitigate the underlying causes of these findings.

## Notes

Supplement 1. **eTable 1.** Comparison of the linked and unlinked vital statistics-patient discharge data **eTable 2.** Source of variables **eTable 3.** Adjusted risk ratios for adverse maternal and neonatal outcomes using either birth certificate only or *ICD-9/10* codes only **eTable 4.** Adjusted risk of maternal and neonatal outcomes in individuals with forceps delivery and vacuum delivery **eTable 5.** Adjusted risk of maternal and neonatal outcomes in nulliparous and multiparous individuals

Supplement 2.
Data Sharing Statement
