# Supplementary material for: Obstetric Outcomes by Hospital Volume of Operative Vaginal Delivery
Source: JAMA Netw Open. 2025 Jan 6;8(1):e2453292. doi: 10.1001/jamanetworkopen.2024.53292 (PMC11704972; doi:10.1001/jamanetworkopen.2024.53292)
Supplement: Supplement 1. — eTable 1. Comparison of the linked and unlinked vital statistics-patient discharge data eTable 2. Source of variables eTable 3. Adjusted risk ratios for adverse maternal and neonatal outcomes using either birth certificate only or ICD-9/10 codes only eTable 4. Adjusted risk of maternal and neonatal outcomes in individuals with forceps delivery and vacuum delivery eTable 5. Adjusted risk of maternal and neonatal outcomes in nulliparous and multiparous individuals [file jamanetwopen-e2453292-s001.pdf]

Supplemental Online Content

Willy AS, Hersh AR, Garg B, Caughey AB. Obstetric outcomes by hospital volume of operative vaginal delivery. *JAMA Netw Open*. 2025;8(1):e2453292.
doi:10.1001/jamanetworkopen.2024.53292

eTable 1. Comparison of the linked and unlinked vital statistics-patient discharge data
eTable 2. Source of variables
eTable 3. Adjusted risk ratios for adverse maternal and neonatal outcomes using either birth certificate only or *ICD-9/10* codes only
eTable 4. Adjusted risk of maternal and neonatal outcomes in individuals with forceps delivery and vacuum delivery
eTable 5. Adjusted risk of maternal and neonatal outcomes in nulliparous and multiparous individuals

This supplemental material has been provided by the authors to give readers additional information about their work.

© 2025 Willy AS et al. *JAMA Network Open*.

eTable 1. Comparison of the linked and unlinked vital statistics-patient discharge data

	Unlinked (n= 362,458)	Linked (n= 5,841,342)	Total (n= 6,203,800)
Race/ethnicity			
Non-Hispanic white	25.6%	26.7%	26.6%
Non-Hispanic Black	5.4%	5.0%	5.1%
Hispanic	49.2%	50.1%	50.0%
Asian/Pacific Islanders	11.5%	11.3%	11.3%
AIAN	0.3%	0.3%	0.3%
Other/Multiracial	7.9%	6.7%	6.7%
Maternal age (Years)			
<20	5.8%	6.2%	6.2%
20-34	71.4%	73.2%	73.1%
>=35	22.8%	20.6%	20.7%
Education			
High school or less	39.8%	42.8%	42.6%
Some college	60.2%	57.2%	57.4%
Pre-pregnancy BMI			
Underweight	3.5%	3.8%	3.8%
Normal weight	45.7%	46.8%	46.7%
Overweight	27.0%	26.4%	26.4%
Obese	23.8%	23.0%	23.1%
Insurance			
Private	54.6%	48.4%	48.7%
Public	45.4%	51.6%	51.3%
Parity			
Multiparous	60.4%	61.1%	61.1%
Nulliparous	39.6%	38.9%	38.9%

© 2025 Willy AS et al. *JAMA Network Open*.

eTable 2. Source of variables

Variable	Data source	Notes/*ICD-9* codes	*ICD-10* codes
Race and ethnicity	Vital statistics		
Age	Vital statistics		
Education	Vital statistics		
Pre-pregnancy body mass index	Vital statistics	Calculated from height and pre-pregnancy weight	
Insurance	Vital statistics		
Parity	Vital statistics		
Operative vaginal delivery	Vital statistics	Diagnosis codes (669.51) Procedure codes (72.0, 72.1, 72.2, 72.3, 72.4, 72.6, 72.7)	Diagnosis codes (O66.5) Procedure codes (10D07Z3, 10D07Z4, 10D07Z5, 10D07Z6, 0W8NXZZ, 10S07ZZ
Obstetric anal sphincter injuries	Vital statistics	Diagnosis codes (664.2,664.3, 664.6)	Diagnosis codes (O70.2, O70.3, O70.4)
Cervical lacerations		665.3	O71.3
Postpartum hemorrhage		666.0, 666.1, 666.2, 666.3	O72.x
Shoulder dystocia	Vital statistics	660.4	O66.0
NICU admission	Vital statistics		
Subgaleal hemorrhage		767.0	P12.2
Intracranial hemorrhage		772.1, 772.2, 767.0	P52
Facial nerve injury		951.4, 767.5	S04.5, P11.3
Fractures		800.x, 801.x, 812.x, 767.2	S02.0, S02.1, S42.2, S42.3, S42.4, P13.4
Brachial plexus injury		767.6, 953.4	P14.0, P14.1, P14.3, S14.3

© 2025 Willy AS et al. *JAMA Network Open*.

eTable 3. Adjusted risk ratios for adverse maternal and neonatal outcomes using either birth certificate only or *ICD-9/10* codes only

	Using only birth certificate			Using only *ICD-9/10* codes		
	Low volume hospitals	Medium volume hospitals	High volume hospitals	Low volume hospitals	Medium volume hospitals	High volume hospitals
Maternal outcomes						
Obstetric anal sphincter injury	1.21 (1.02, 1.43)	1.07 (0.92,1.24)	Reference	1.37 (1.13, 1.65)	1.14 (0.94, 1.37)	Reference
Cervical lacerations	1.00 (0.65, 1.55)	1.19 (0.84, 1.69)	Reference	1.26 (0.91, 1.74)	1.20 (0.88, 1.63)	Reference
Postpartum hemorrhage	0.90 (0.68, 1.20)	0.98 (0.76, 1.28)	Reference	1.42 (1.07, 1.88)	1.14 (0.86, 1.52)	Reference
Neonatal outcomes						
Shoulder dystocia	0.88 (0.76, 1.02)	0.93 (0.80, 1.09)	Reference	1.34 (1.13, 1.57)	1.24 (1.06, 1.45)	Reference
NICU admission	1.17 (1.00, 1.37)	1.09 (0.95, 1.26)	Reference	1.13 (0.96, 1.32)	1.11 (0.94, 1.30)	Reference
Subgaleal hemorrhage	1.21 (0.64, 2.31)	1.39 (0.82, 2.34)	Reference	2.84 (1.60, 5.02)	2.03 (1.13, 3.68)	Reference
Intracranial hemorrhage	1.23 (0.58, 2.60)	0.93 (0.51, 1.70)	Reference	1.12 (0.56, 2.21)	1.24 (0.69, 2.24)	Reference
Facial nerve injury	1.30 (0.89, 2.84)	1.24 (0.53, 2.91)	Reference	1.67 (0.71, 3.94)	1.13 (0.58, 2.21)	Reference
Fracture	1.29 (0.99, 1.67)	0.88 (0.70, 1.11)	Reference	1.33 (1.05, 1.70)	1.28 (1.03, 1.58)	Reference
Brachial plexus injury	1.05 (0.73, 1.53)	0.88 (0.65, 1.18)	Reference	1.81 (1.37, 2.41)	1.36 (1.03, 1.81)	Reference

© 2025 Willy AS et al. *JAMA Network Open*.

eTable 4. Adjusted risk of maternal and neonatal outcomes in individuals with forceps delivery and vacuum delivery

	Forceps (n=15,006)		Vacuum (n=230,613)	
	Percentage	Adjusted risk ratio (95% CI)	Percentage	Adjusted risk ratio (95% CI)
Maternal outcomes				
Obstetric anal sphincter lacerations				
Low-OVD volume	22.03%	1.40 (1.15-1.71)	13.24%	1.35 (1.15-1.59)
Medium-OVD volume	20.68%	1.14 (0.94-1.39)	12.05%	1.08 (0.91-1.27)
High OVD-volume	19.92%	Reference	10.62%	Reference
Cervical Lacerations				
Low-OVD volume	0.74%	1.83 (0.86-3.90)	0.29%	1.40 (0.99-1.96)
Medium-OVD volume	0.61%	1.46 (0.76-2.81)	0.24%	1.06 (0.80-1.40)
High OVD-volume	0.48%	Reference	0.21%	Reference
Post-partum hemorrhage				
Low-OVD volume	8.54%	1.03 (0.67-1.57)	5.40%	1.37 (1.01-1.86)
Medium-OVD volume	6.04%	0.83 (0.57-1.19)	4.26%	1.05 (0.76-1.44)
High OVD-volume	8.19%	Reference	3.80%	Reference
Neonatal outcomes				
Shoulder dystocia				
Low-OVD volume	3.55%	1.25 (0.88-1.78)	3.69%	1.19 (1.02-1.39)
Medium-OVD volume	2.48%	0.96 (0.69-1.35)	3.61%	1.12 (0.97-1.28)
High OVD-volume	2.40%	Reference	3.08%	Reference
NICU admission				
Low-OVD volume	12.40%	1.45 (1.16-1.81)	10.71%	1.16 (0.99-1.35)
Medium-OVD volume	9.89%	1.17 (0.97-1.41)	9.65%	1.09 (0.95-1.26)
High OVD-volume	8.02%	Reference	9.25%	Reference
Subgaleal hemorrhage				
Low-OVD volume	0.23%	3.88 (1.39-10.81)	0.34%	2.61 (1.57-4.33)
Medium-OVD volume	0.19%	2.78 (1.09-7.04)	0.22%	1.63 (0.99-2.67)
High OVD-volume	0.08%	Reference	0.12%	Reference
Intracranial hemorrhage				
Low-OVD volume	0.06%	2.92 (0.37-22.74)	0.02%	0.71 (0.32-1.58)
Medium-OVD volume	0.07%	2.98 (0.42-21.16)	0.05%	1.36 (0.76-2.43)
High OVD-volume	0.03%	Reference	0.03%	Reference
Facial nerve palsy				
Low-OVD volume	0.52%	1.53 (0.63-3.70)	0.02%	1.91 (0.81-4.46)
Medium-OVD volume	0.42%	1.27 (0.60-2.70)	0.01%	1.12 (0.43-2.89)
High OVD-volume	0.33%	Reference	0.01%	Reference
Fractures				
Low-OVD volume	0.32%	0.58 (0.28-1.21)	0.58%	1.44 (1.13-1.81)

© 2025 Willy AS et al. *JAMA Network Open*.

Medium-OVD volume	0.64%	1.17 (0.63-2.17)	0.51%	1.27 (1.03-1.58)
High OVD-volume	0.52%	Reference	0.41%	Reference
Brachial plexus injury				
Low-OVD volume	0.54%	1.26 (0.51-3.12)	0.40%	1.69 (1.26-2.27)
Medium-OVD volume	0.61%	1.69 (0.75-3.82)	0.28%	1.23 (0.92-1.62)
High OVD-volume	0.30%	Reference	0.21%	Reference

© 2025 Willy AS et al. *JAMA Network Open*.

eTable 5. Adjusted risk of maternal and neonatal outcomes in nulliparous and multiparous individuals

	Nulliparous (n=202,925)		Multiparous (n=103,788)	
	Percentage	Adjusted risk ratio (95% CI)	Percentage	Adjusted risk ratio (95% CI)
Maternal outcomes				
Obstetric anal sphincter lacerations				
Low-OVD volume	15.63%	1.34 (1.13-1.59)	5.45%	1.50 (1.12-2.02)
Medium-OVD volume	14.25%	1.09 (0.92-1.28)	4.57%	1.11 (0.84-1.46)
High OVD-volume	12.37%	Reference	3.87%	Reference
Cervical Lacerations				
Low-OVD volume	0.33%	1.29 (0.90-1.84)	0.27%	1.52 (0.94-2.46)
Medium-OVD volume	0.26%	0.95 (0.68-1.34)	0.23%	1.26 (0.86-1.85)
High OVD-volume	0.25%	Reference	0.19%	Reference
Post-partum hemorrhage				
Low-OVD volume	6.06%	1.31 (0.97-1.77)	4.22%	1.57 (1.17-2.12)
Medium-OVD volume	4.68%	0.99 (0.73-1.36)	3.24%	1.18 (0.88-1.56)
High OVD-volume	4.37%	Reference	2.58%	Reference
Neonatal outcomes				
Shoulder dystocia				
Low-OVD volume	3.00%	1.25 (1.05-1.49)	5.46%	1.37 (1.16-1.63)
Medium-OVD volume	2.80%	1.11 (0.95-1.30)	4.91%	1.22 (1.03-1.44)
High OVD-volume	2.30%	Reference	3.73%	Reference
NICU admission				
Low-OVD volume	10.31%	1.15 (0.98-1.36)	9.63%	1.17 (1.01-1.34)
Medium-OVD volume	9.61%	1.10 (0.95-1.29)	8.74%	1.11 (0.97-1.27)
High OVD-volume	8.99%	Reference	8.21%	Reference
Subgaleal hemorrhage				
Low-OVD volume	0.36%	2.76 (1.63-4.69)	0.09%	1.49 (0.69-3.19)
Medium-OVD volume	0.22%	1.65 (0.96-2.83)	0.08%	1.37 (0.68-2.74)
High OVD-volume	0.12%	Reference	0.05%	Reference
Intracranial hemorrhage				
Low-OVD volume	0.02%	0.52 (0.19-1.41)	0.03%	1.43 (0.49-4.20)
Medium-OVD volume	0.05%	1.52 (0.78-2.97)	0.02%	0.91 (0.31-2.63)
High OVD-volume	0.03%	Reference	0.02%	Reference
Facial nerve palsy				
Low-OVD volume	0.06%	1.82 (0.91-3.65)	0.03%	1.14 (0.33-3.92)
Medium-OVD volume	0.04%	1.07 (0.54-2.09)	0.01%	0.47 (0.07-3.19)
High OVD-volume	0.03%	Reference	0.02%	Reference
Fractures				
Low-OVD volume	0.49%	1.31 (0.98-1.74)	0.61%	1.50 (1.12-2.01)
Medium-OVD volume	0.47%	1.25 (0.98-1.60)	0.52%	1.32 (0.99-1.74)
High OVD-volume	0.36%	Reference	0.41%	Reference

© 2025 Willy AS et al. *JAMA Network Open*.

Brachial plexus injury				
Low-OVD volume	0.37%	1.67 (1.19-2.35)	0.48%	1.71 (1.23-2.36)
Medium-OVD volume	0.27%	1.26 (0.91-1.75)	0.36%	1.37 (0.97-1.93)
High OVD-volume	0.19%	Reference	0.23%	Reference

© 2025 Willy AS et al. *JAMA Network Open*.
