# Supplementary material for: Obstetric Outcomes by Hospital Volume of Operative Vaginal Delivery
Source: JAMA Netw Open. 2025 Jan 6;8(1):e2453292. doi: 10.1001/jamanetworkopen.2024.53292 (PMC11704972; doi:10.1001/jamanetworkopen.2024.53292)
Supplement: Supplement 2. — Data Sharing Statement [file jamanetwopen-e2453292-s002.pdf]

Data Sharing Statement

Willy. Obstetric Outcomes by Hospital Volume of Operative Vaginal Delivery. *JAMA Netw Open*. Published January 06, 2025. doi:10.1001/jamanetworkopen.2024.53292

Data
Data available: No

Additional Information
Explanation for why data not available: Data used is a part of the California Data set, no individual patient data available
